# Evaluating fruit quality of pear varieties with different textures after diverse cold-storage times

**DOI:** 10.1016/j.fochx.2025.103357

**Published:** 2025-12-01

**Authors:** Chen Yin, Luming Tian, Hongliang Huo, Jing Li, Xingguang Dong, Ying Zhang, Dan Qi, Jiayu Xu, Chao Liu, Yufen Cao

**Affiliations:** a Institute of Pomology, Chinese Academy of Agricultural Sciences, Xingcheng, 125100, China; b Key Laboratory of Germplasm Resources Utilization of Horticultural Crops, Ministry of Agriculture and Rural Affairs, Xingcheng, 125100, China

**Keywords:** Pear, Cold-storage, Shelf-life, Sugars, Organic acids, Texture

## Abstract

Cold-storage is a widely-used technique for effectively maintaining pear quality. In this study, ultraperformance liquid chromatography tandem mass spectrometry (UPLC-MS/MS) was employed to determine sugar and organic acid components in pears of different textures after different cold-storage times, while texture analyzer was adopted to assess the texture traits of pear fruits. Different cold-storage times affected the quality of fruits from different pear varieties, whereas extended cold-storage led to quality deterioration. After cold-storage, the difference in sucrose between soft-flesh pears and crispy-flesh pears decreased and eventually faded with extended storage. Based on principal component analysis (PCA), Nanguoli, Jingbaili, Ruanerli, Korla pear, Chili and Yali achieved the highest fruit quality after 2 months of cold-storage, while Dangshan Suli and Shuihongxiao peaked after 4 months. This work provides a better strategy for determining suitable cold-storage time and shelf-life for pear varieties by comprehensively considering sugars, organic acids and texture variation.

## Highlights

• Clarified the variations in fruit quality traits induced by cold-storage.
• Proposed a method for determining the optimal storage scheme.
• Identified sugars, organic acids and texture as the powerful quality trinity.

## Graphical abstract

**Figure:**
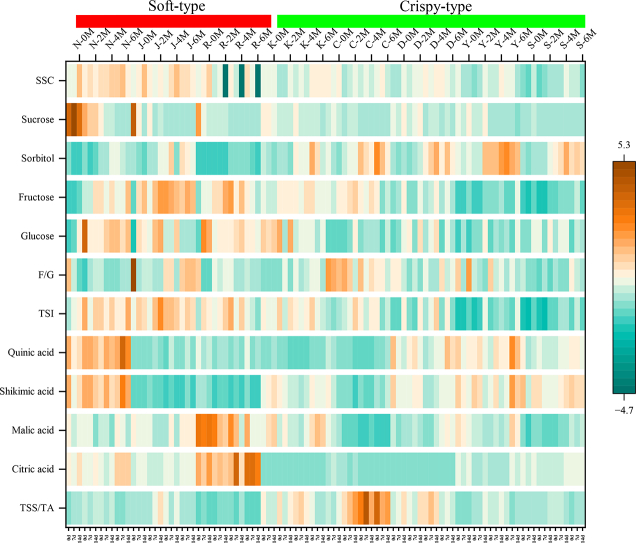

## 1 Introduction

Fruit quality is a comprehensive concept that includes physical characters (size, shape and firmness), sensory characters (sweetness, acidity, texture and aroma), appearance characters (fruit surface smoothness and color), and nutrition related to food safety (Xu et al., 2022). All of these aspects may affect consumers' purchase decisions and fruit sales (Xu et al., 2022). Cold-storage helps maintain the great flavor and prolong quality of pears by reducing fruit respiration, oxidase activity, and nutrient consumption (Yi et al., 2020). It is not only the simplest and most effective method, but also the most widely adopted method in fruit commerce (Híc et al., 2023). Cold-storage ensures a prolonged supply of fresh pears in the market, avoids gluts and transportation bottlenecks during peak seasons, and thus provides remunerative prices for the farmers (Sinha et al., 2022).

The intrinsic quality of fruits mainly depends on the proportion of soluble sugars, which is influenced by various factors such as genetics, the natural environment and cultivation measures (Xu et al., 2022). These factors form a complex metabolic regulatory network that affects enzyme activity, genetic expression, sugar content, and sugar metabolism (Xu et al., 2022). Low-temperature storage delays the loss of soluble sugars and help maintain nutritional quality by reducing the respiration rate and metabolic activity of fruits (Cheng et al., 2021). Maintaining high levels of soluble sugars during cold-storage also helps protect plants from low-temperature stress, as they act as scavengers of reactive oxygen species, osmotic pressure regulators, cryoprotectants, and signaling molecules (Wang et al., 2024). Organic acids also play a crucial role in fruit flavor and overall sensory quality. Malic acid, an intermediate in the tricarboxylic acid cycle, is an important storage carbon molecule and pH regulator (Liu et al., 2016).

Fruits firmness is another important quality attribute, as excessive softening reduces weight and lowers fresh fruit value (Mao et al., 2022). Changes in cell wall structure affect cell division or separation and determine the texture of fruit (Brummell and Harpster, 2001). During cold-storage and shelf-life, fruits continue to ripen, which involves extensive modification of the cell wall polysaccharides by a variety of ripening-related enzymes (Wang et al., 2013), and the resulting structural changes affect cell separation, thereby determining fruit texture (Brummell and Harpster, 2001).

Generally speaking, flavor, organic acids, texture and juiciness, are key factors influencing the overall taste of the fruit (Kouassi et al., 2009). Previous research has found that each pear variety requires a customized strategy during storage to achieve optimal sensory attributes (Híc et al., 2023). Our previous research has already indicated that a combination of sugars, organic acids and texture has the potential to be a good indicator for evaluating pear maturity and quality (Yin et al., 2025). The aim of this study was to explore the potential of sugars, organic acids and texture in the quality evaluation of pears after cold-storage. Our findings could provide reference for determining the suitable cold-storage time and shelf-life for different pear varieties. To achieve this goal, we selected pear varieties of different texture; measured the variation in sugars, organic acids and flesh texture during the 0-14 day (d) shelf-life after different cold-storage times; conducted a comprehensive analysis to evaluate the fruits quality; and speculated on suitable cold-storage time and shelf-life.

## 2 Materials and methods

### 2.1 Materials

**Fig. 1:**
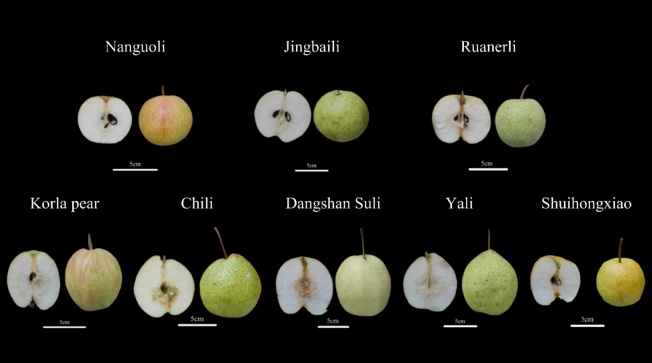
Picture of pear fruits picked at 146 days after flower bloom (DAFB).

Three soft-flesh pear varieties (Cao and Zhang, 2020), Nanguoli (*Pyrus ussuriensis* Maxim.), Jingbaili (*Pyrus ussuriensis* Maxim.), Ruanerli (*Pyrus ussuriensis* Maxim.), and five crispy-flesh pear varieties (Cao and Zhang, 2020), Korla Pear (*Pyrus sinkiangensis* Yu.), Chili (*Pyrus bretschneideri* Rehd.), Dangshan Suli (*Pyrus bretschneideri* Rehd.), Yali (*Pyrus bretschneideri* Rehd.), Shuihongxiao (*Pyrus bretschneideri* Rehd.), were selected for this experiment. All samples were collected from the National Germplasm Repository of Pear and Apple (Xingcheng, China) at September 20th, 2023 (Fig. 1). We placed the pear samples of each variety in separate food-grade plastic bags and stored them in a RA storage room with independent air-cooled units at the Fruit Storage and Processing Research Centre (Xingcheng, China). Cold-storage (0 ± 1 °C) periods of 0 month (M), 2 M, 4 M and 6 M were applied to all samples, followed by a shelf-life period of 0 days (d), 7 d and 14 d, at room temperature.

### 2.2 Extraction and determination of sugars and organic acids

About 100 g of pears were ground into a powder in liquid nitrogen by a SPEX sample prepsystem and stored at −80 °C for the determination of soluble sugars and organic acids. Three repetitions were set for each sample. Sugars and organic acids were extracted according to a method reported previously (Zhang et al., 2020 & Zhang et al., 2022), and stored at −20 °C.

To determine sugar compositions, the UPLC-MS/MS system with a Waters ACQUITY UPLC BEH Amide column (1.7 μm × 100 mm × 2.1 mm) was used. The A1 mobile phase consisted of 85 % (*V*/V) acetonitrile (100 %) (Pennsylvania, Beijing, China) and 15 % (V/V) ammonia solution (0.1 %) (J&K, Beijing, China), the B1 mobile phase consisted of acetonitrile (100 %) and the flow rate was 0.3 mL/min. To determine organic acid compositions, the UPLC-MS/MS system with a Waters Atlantis™ Premier BEH C18 AX column (1.7 μm × 100 mm × 2.1 mm) was used. The A1 mobile phase consisted of formic acid (0.5 %) (ACS, Shanghai, China), the B1 mobile phase consisted of acetonitrile (100 %), and the flow rate of 0.3 mL/min.

For the preparation of sugars standard solution and organic acid standard solution, proportions of fructose, sorbitol, sucrose and glucose (Sigma, Shanghai, China) were 50–50–25-25 mg/L, and proportions of malic acid, citric acid, quinic acid, shikimic acid, succinic acid and fumaric acid (Chemservice, Beijing, China) were 100–100–40-20-4-4 mg/L. Subsequently, the sugar standard solution was diluted 5 times and 25 times, the organic acid standard solution was diluted 4 times and 20 times. Data processing and instrument control were carried out using MassLynx V4.1. Individual sugars and organic acids were identified and quantified by comparison with the retention time and peak areas of individual sugar standards and individual organic acid standards (Supplementary Table S1).

The individual sugars (sucrose, fructose, glucose and sorbitol) were calculated using appropriate standards and expressed in g/kg of fresh fruit weight (FW). The individual organic acids (citric acid, malic acid, quinic acid, tartaric acid, shikimic acid, succinic acid and fumaric acid) were calculated using appropriate standards and expressed in mg/g FW. No effective peaks were detected for tartaric acid, succinic acid or fumaric acid. Total soluble sugars (TSS) and total organic acids (TA) were obtained by summing the concentrations of the individual components. Research showed that pear fruits with high F/G have a sweeter taste (Yin et al., 2024). The fructose to glucose ratio (F/G) was calculated according to fructose and glucose. The sweetness levels of each sugar component were different. The relative sweetness levels of the sugar components were adopted from Jia et al. (2023), with sucrose set as the standard (1.0), followed by sorbitol (0.6), glucose (0.75), and fructose (1.7). Total sweetness index (TSI) was based on the following equation (He et al., 2022):

TSI = C_suc_ × 1 + C_sor_ × 0.6 + C_glu_ × 0.75 + C_fru_ × 1.7.

where C_suc_, C_sor_, C_glu_ and C_fru_ were the content of sucrose, sorbitol, glucose and fructose, respectively.

### 2.3 Determination of SSC and texture traits

Soluble solid contents (SSC) were measured by using a PR-101α sugar analyzer from ATAGO Company in Japan, with ten randomly selected samples of each pear variety.

The texture characteristics were determined using the texture profile analysis (TPA) method of the TMS-TOUCH food property analyzer from Food Technology Corporation in USA. The method was based on Qiu et al. (2021) with modifications. The TPA method used a P/35 (diameter 75 mm) probe for the experiment, with parameter settings as follows: the height of the probe rising to the surface of the sample was 10 mm, the deformation percentage was 50 %, the detection speed was 30 mm/min and the initial force was 1 N. Measurements were carried out on ten randomly selected fruits. The indicators based on the TPA for measuring texture traits were flesh firmness, fracture, adhesiveness, cohesiveness, springiness, gumminess, chewiness and firmness1 to firmness2 ratio (F1/F2). F1/F2 represents the softening speed of the flesh to a certain extent.

### 2.4 Statistical analyses

The analysis of variance (ANOVA) established differences in mean values, and these were tested by Tukey's test at a significance level of *p* < 0.05, which was performed via SPSS 25. Pearson correlation analysis, the cluster analysis and principal component analysis (PCA) were used via Origin 2024. Orthogonal partial least squares discriminant analysis (OPLS-DA) was used via online software MetaboAnalyst 6.0 (https://www.metaboanalyst.ca).

## 3 Results and discussion

### 3.1 Variation of qualitative character in pear fruit after different cold storage times

#### 3.1.1 Sugar components of pear fruits

**Fig. 2:**
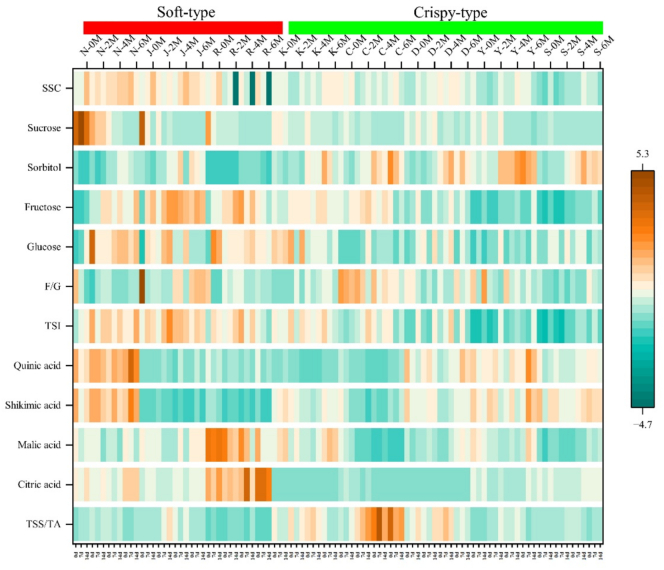
Variation of SSC, sugar and organic acid components in pear varieties at shelf-life of 0–14 d after 0, 2, 4, 6 M of cold-storage. N, J, R, K, C, D, Y, S represent Nanguoli, Jingbaili, Ruanerli, Korla pear, Chili, Dangshan Suli, Yali and Shuihongxiao, respectively. The original data are presented in Table S2.

Cold-storage led to a decrease in SSC, sucrose and F/G, while fructose, glucose and sorbitol increased (Fig. 2). During the 0–4 M of cold-storage, sucrose and glucose contents decreased, whereas SSC, sorbitol, fructose, F/G, TSS/TA and TSI increased (Fig. 2). In soft-flesh pears, significant differences were observed in the variation in sucrose, sorbitol, fructose and TSI across different cold-storage times, with the variations in sucrose and fructose were concentrated on the 0–2 M of cold-storage (Table 1). In crispy-flesh pears, significant differences were observed in the variation in SSC, sucrose and sorbitol after different cold-storage times (Table 1). The significance of glucose variation depended on the fruit texture (Table 1). Research has proved the metabolic process of converting sucrose into glucose and fructose during cold-storage, which is called cold induced sweetening, and longer cold-storage time may lead to higher sugar content (Dias et al., 2022). Additionally, low-temperature storage may also weaken glycolysis and reduce the rate of glucose decomposition (Cheng et al., 2021). With the extension of cold-storage time from 4 to 6 M, SSC, F/G and sorbitol continued to increase in most pears, while other sugar components, TSS/TA and TSI decreased (Fig. 2). Under cold-storage conditions, the sugar content in pears initially increased but eventually decreased toward the end of the period. This trend may be attributed to the hydrolysis of complex insoluble polysaccharides into simple soluble monosaccharides and disaccharides, followed by a decrease caused by the complete breakdown of starch and carbohydrate utilization during respiration in the later storage stages (Sinha et al., 2022). During the 0–14 d shelf-life following different cold-storage times, the variation of sugar components varied among different pear varieties (Fig. 2). In soft-flesh pears stored for 0 M cold-storage, the variation differences in SSC, F/G and TSI during the 0–14 d shelf-life reached significant levels, though these significances vanished after cold-storage treatment (Table 2). It was worth mentioning that the difference of sorbitol between crispy-flesh pears and soft-flesh pears reached a significant level during the 0–14 d shelf life after 0, 4, and 6 M cold-storage (Table 2).

**Table 1:** ANOVA of sugar and organic acid components in soft-flesh pears and crispy-flesh pears during 0–6 M of cold-storage.

Significance											
Cold storage time			0 M	2 M	4 M	6 M		0 M	2 M	4 M	6 M
	Total	Soft-flesh pears					Crispy-flesh pears				
SSC	***	0.313	14.28 ± 1.84	13.76 ± 0.46	14.77 ± 1.35	14.80 ± 0.70	*	12.22 ± 0.54ab	11.74 ± 0.91b	12.64 ± 0.40ab	12.82 ± 0.85a
Sucrose	***	***	0.55 ± 0.220a	0.23 ± 0.054b	0.10 ± 0.035b	0.06 ± 0.007b	***	0.17 ± 0.011a	0.12 ± 0.035ab	0.09 ± 0.017bc	0.07 ± 0.015c
Sorbitol	***	*	0.16 ± 0.039b	0.20 ± 0.026ab	0.30 ± 0.047ab	0.32 ± 0.052a	***	0.29 ± 0.032b	0.33 ± 0.053b	0.55 ± 0.037a	0.58 ± 0.091a
Fructose	***	**	0.74 ± 0.133b	0.99 ± 0.136a	1.08 ± 0.095a	0.98 ± 0.035a	0.417	0.70 ± 0.061	0.75 ± 0.060	0.80 ± 0.040	0.76 ± 0.012
Glucose	**	0.247	0.54 ± 0.307	0.76 ± 0.072	0.70 ± 0.106	0.61 ± 0.021	0.225	0.43 ± 0.045	0.55 ± 0.157	0.48 ± 0.085	0.45 ± 0.055
F/G	0.485	0.48	2.06 ± 1.40	1.4 ± 0.15	1.62 ± 0.34	1.77 ± 0.06	0.296	1.96 ± 0.17	1.51 ± 0.32	1.76 ± 0.31	1.76 ± 0.23
TSI	***	*	2.32 ± 0.226b	2.60 ± 0.196ab	2.63 ± 0.156a	2.37 ± 0.023ab	0.194	1.86 ± 0.135	2.00 ± 0.272	2.13 ± 0.122	2.05 ± 0.041
Quinic acid	0.385	0.782	0.3 ± 0.05	0.31 ± 0.03	0.29 ± 0.07	0.38 ± 0.06	0.15	0.28 ± 0.06	0.26 ± 0.03	0.22 ± 0.02	0.33 ± 0.05
Shikimic acid	0.204	0.964	0.08 ± 0.01	0.08 ± 0.01	0.07 ± 0.01	0.08 ± 0.01	*	0.11 ± 0.02a	0.09 ± 0.01ab	0.08 ± 0.002b	0.11 ± 0.01ab
Malic acid	***	0.299	3.32 ± 0.27	2.58 ± 0.56	2.33 ± 0.59	2.5 ± 0.51	*	1.92 ± 0.18ab	1.45 ± 0.11ab	1.36 ± 0.18b	2.09 ± 0.28a
Citric acid	***	0.234	3.34 ± 0.53	2.65 ± 0.08	3.2 ± 1.03	4.68 ± 0.22	0.467	1.03 ± 0.04	0.72 ± 0.09	0.62 ± 0.13	0.82 ± 0.09
TSS/TA	***	0.101	32.61 ± 4.93	47.23 ± 7.89	45.13 ± 8.48	29.71 ± 2.31	*	52.86 ± 6.78b	76.30 ± 14.52ab	102.61 ± 16.92a	74.75 ± 12.38ab

Note: * represents *p* *<* *0.05*, ** represents *p* *<* *0.01*, *** represents *p* *<* *0.001*, different lowercase letters in the same line indicates significant differences of soft-flesh pears or crispy-flesh pears at different cold-storage periods (*p* *<* *0.05*). The total column indicates the degree of difference in traits variation between two types of pears during 0–6 M of cold storage.

**Table 2:** ANOVA of quality traits in soft-flesh pears and crispy-flesh pears after different cold-storage times during a 0–14 d shelf-life.

	Significance											
Cold storage time	0 M			2 M			4 M			6 M		
	Total	Soft type	Crispy type	Total	Soft type	Crispy type	Total	Soft type	Crispy type	Total	Soft type	Crispy type
SSC	***	***	0.401	**	0.542	*	**	0.058	0.428	**	0.489	0.052
Sucrose	*	0.461	0.880	0.269	0.763	0.226	0.581	0.411	0.553	0.777	0.732	0.461
Sorbitol	**	0.448	0.250	0.077	0.871	0.250	**	0.731	0.616	**	0.705	0.082
Fructose	0.415	0.343	0.462	0.057	0.129	0.672	**	0.288	0.668	0.073	0.884	0.961
Glucose	0.074	0.063	0.816	0.104	0.850	*	**	0.121	0.100	0.193	0.970	0.271
F/G	0.075	*	0.852	0.639	0.713	0.346	0.263	0.309	0.191	0.846	0.981	0.090
TSI	*	*	0.518	*	0.410	0.148	**	0.154	0.419	0.387	0.988	0.921
Quinic acid	0.683	0.716	0.328	0.956	0.948	0.805	0.754	0.745	0.833	0.952	0.912	0.703
Shikimic acid	0.253	0.850	0.104	0.899	0.965	0.362	0.964	0.892	0.983	0.906	0.928	0.907
Malic acid	0.196	0.927	0.795	0.087	0.613	0.808	0.064	0.473	0.570	0.614	0.170	0.727
Citric acid	**	0.750	0.988	*	0.991	0.902	*	0.669	0.850	**	0.986	0.952
TSS/TA	0.220	0.669	0.627	0.255	0.798	0.411	0.170	0.545	0.677	0.394	0.921	0.804
Fracture	–	–	–	***	***	0.584	***	*	0.083	***	*	0.341
Flesh firmness	–	–	–	***	***	0.587	**	*	0.251	**	0.056	0.316
F1/F2	–	–	–	0.464	0.477	0.495	0.974	0.964	0.507	0.741	0.651	0.537
Adhesiveness	–	–	–	0.525	0.232	0.764	0.061	*	0.165	0.056	0.660	0.483
Cohesiveness	–	–	–	**	0.095	0.068	**	*	0.692	*	0.141	0.933
Springiness	–	–	–	***	***	*	***	**	0.110	***	*	0.854
Gumminess	–	–	–	***	**	0.906	***	*	*	***	*	0.319
Chewiness	–	–	–	***	**	0.559	***	*	*	***	*	0.482

Note: * represents *p* *<* *0.05*, ** represents *p* *<* *0.01*, *** represents *p* *<* *0.001*, − represents the data was empty.

Cold-storage significantly reduced sucrose content in pears. As cold-storage prolonged to 4 M, the difference in sucrose diminished between soft-flesh pears classified as high-sucrose type and crispy-flesh pears classified as low-sucrose type (Fig. 2). This decrease commenced at the beginning of the storage period in soft-flesh pears. Although cold-storage reduced sucrose in crispy-flesh pears, short-term exposure increased sucrose content in some crispy-flesh pear varieties, with the decline occurred on the 2–4 M of cold-storage (Fig. 2). According to Table 2, the *p* value represented the difference of sucrose in pears during the shelf life of 0–14 d after 0 M cold-storage was 0.013, reaching a significant level. However, the *p* values gradually increased with extended cold-storage time to 0.269 (2 M), 0.581 (4 M), and 0.777 (6 M), respectively, indicating a loss of significance (Table 2). During the development periods, the second highest sugar component in crispy-flesh pears is glucose, classified as low-sucrose types (Yin et al., 2024). In contrast, the second highest sugar component in soft-flesh pears is sucrose, classified as high-sucrose type (Yin et al., 2024). This difference in sucrose degradation patterns under cold-storage might be attributed to the inherent sugar profiles of each texture type. As Wang et al. (2024) found in apples, long-term cold storage may hinder the normal conversion of starch to soluble sugars, thereby exacerbating low-temperature stress and disrupted sugar metabolism.

Soluble sugars, as the main energy source of fruits, play an important role in maintaining cell structure and cold resistance (Wang et al., 2020). A higher sucrose content at 0 °C was associated with enhanced membrane stability and cold tolerance in peach, and because of this, it has been noted to offer a greater protective effect under cold-stress than sorbitol (Wang et al., 2013). The improvement of cold tolerance in loquat is related to the levels of reducing sugars, particularly glucose (Wang et al., 2013), where elevated hexose content and the activity of hexose sensors constitute a part of cold tolerance mechanism (Cao et al., 2013). Likewise, high levels of glucose and fructose are associated with cold tolerance in apricot fruits (Wang et al., 2020). The cold-storage of potato tubers may lead to the accumulation of reducing sugars and the loss of sucrose (Itai and Tanahashi, 2008). The same cold-induced sweetening metabolism process as potato tubers has been observed in pear fruits (Itai et al., 2015). In our study, glucose content increased during the 0–2 M of cold-storage, fructose content continued to increase throughout the 0–4 M cold-storage, and sorbitol maintained a trend of gradually increasing content with prolonged storage time over the 0–6 M cold-storage (Fig. 2). In contrast to peach fruits, the cold tolerance mechanism of pear fruit might be similar to that observed in loquat, apricot and potato tubers. After cold-storage, sucrose decreased rapidly, while the reducing sugar increased. Notably, sorbitol provided a greater protective effect in pears than sucrose, and even a more pronounced effect than fructose and glucose.

#### 3.1.2 Organic acid components of pear fruits

In general, cold-storage reduced the content of organic acid components in pear fruits. The decrease of organic acids in pears was potentially attributed to gluconeogenesis, a process converting organic acid into sugar by metabolism (Pasquariello et al., 2013). Specifically, the quinic acid, shikimic acid and malic acid gradually decreased as cold-storage was extended to 4 M (Fig. 2). However, they increased again when cold-storage was prolonged to 6 M (Fig. 2). The increase of malic acid may be related to the increase of weight loss during longer cold-storage (Dias et al., 2022). Furthermore, the timing of the increase in citric acid between two texture types pears were different. The citric acid of soft-flesh pears increased during the 2–6 M of cold-storage, while that of crispy-flesh pears increased during the 4–6 M of cold-storage (Fig. 2). The significant variation in citric acid depended on the fruit texture (Table 1). There were significant differences in the variation of shikimic acid and malic acid in crispy-flesh pears across different cold-storage times, while those in soft-flesh pears did not reach a significant level (Table 1). Within 0–14 d shelf-life, cold-storage for up to 4 M reduced the difference in citric acid between soft-flesh pears and crispy-flesh pears (Table 2). As the cold-storage was extended to 6 M, the difference increased (Table 2). During 6 M of cold-storage, the content of organic acid components in pears increased, which indicated that prolonged cold-storage ultimately led to their accumulation. The accumulation of weak acids such as citric acid and malic acid means that vacuoles are buffered (Holcroft and Kader, 1999). Organic acids are the main substrates for respiration, and the increase in apple acid value is probably related to the possible respiratory slowdown that might occur during longer cold-storage (Dias et al., 2022). From a metabolic perspective, citrate accumulation in storage may result from the outflow of vacuole storage (Flaherty et al., 2018).

#### 3.1.3 Texture traits of pear fruits

During the 2–6 M of cold-storage, the fracture, flesh firmness, adhesiveness, gumminess and chewiness of both soft-flesh pears and crispy-flesh pears exhibited similar tendency, gradually decreasing with prolonged cold-storage time (Table S3). The flesh firmness of cold-stored soft-flesh pears decreased rapidly during the 0–7 d shelf-life, with a decline rate exceeding 80 % (Table S3). However, the flesh firmness of those without cold-storage began to decline rapidly from the 7–14 d shelf-life (Yin et al., 2025), indicating that cold-storage accelerated the onset of the softening process. Low-temperature regulation has been shown to induce transcription factors related to fruit softening (Dias et al., 2022). The differences in the fracture, flesh firmness, springiness, gumminess and chewiness of soft-flesh pears during the shelf-life gradually diminished with extended cold-storage time, while most differences remained statistically significant (Table 2). Most of the differences in texture traits in crispy-flesh pears during shelf-life were not significant (Table 2). It is demonstrated that cold-storage had a significantly greater impact on the texture of soft-flesh pears than on crispy-flesh pears. Fruit firmness is closely related to the structure and composition of cell walls (Pasquariello et al., 2013). Dias (2022) found that a significant decrease in firmness is a characteristic of pear ripening after cold-storage. As fruits continue to mature during cold-storage and shelf-life, extensive modification of cell wall polysaccharides occurs through the action of various ripening related enzymes whose activity is enhanced by ethylene in respiratory climacteric fruits (Pasquariello et al., 2013). The decrease in pear fruit firmness during cold-storage has been attributed to the downregulation of α-1,4-glucan synthase activity (Cheng et al., 2021).

In crispy-flesh pears, glucose is the second most abundant sugar component, which is derived from its comparative levels among other sugar constituents (Yin et al., 2024). However, in terms of actual values, the difference in glucose content between crispy-flesh pears and soft-flesh pears was far less pronounced than the marked disparity observed in their sucrose levels (Table S2). During the 0–6 M of cold-storage, sucrose content changes in both types of pears showed highly significant differences, whereas the variation in glucose did not reach a significant level (Table 1). Additionally, the fructose content in soft-flesh pears exhibited a significant difference, while no significant fluctuation was observed in crispy-flesh pears (Table 1). The decline of pear flesh firmness was significantly correlated with sucrose and glucose (Yin et al., 2025). The cold-induced sweetening involves the conversion of sucrose into glucose and fructose (Dias et al., 2022). The loss of cell wall galactose during early ripening is critical for subsequent changes in fruit firmness and texture (Brummell and Harpster, 2001). The galactose residues on the pectin backbone are broken down into free galactose by β-galactosidase, which may then be converted to galactitol (Song et al., 2023). Cellulose is degraded into glucose by β-glucosidase, and glucose combines with fructose to synthesize sucrose (Song et al., 2023). Sucrose and galactoside alcohols react to produce myo-inositol and raffinose (Song et al., 2023). Based on these, we speculated that the sharp decline in sucrose in soft-flesh pears may partly contribute to the cold-induced sweetening and partly support the earlier initiation of fruit softening.

In summary, the variation tendency of sugar and organic acid components in both soft-flesh pears and crispy-flesh pears during 0–6 M of cold-storage was generally consistent (Fig. 2). During the 0–4 M of cold-storage, SSC, sorbitol, fructose, F/G, TSS/TA, TSI increased (Fig. 2 & Table S3). In contrast, the sucrose, glucose, quinic acid, shikimic acid, malic acid, citric acid, and texture traits decreased (Fig. 2 & Table S3). During the 4–6 M of cold-storage, most sugar components declined, organic acid components increased, and the texture traits continued to decrease (Fig. 2 & Table S3). Sugar and organic acids have a high negative genetic correlation (Kouassi et al., 2009). Organic acids preferentially are used as respiratory substrates during cold-storage, with sugars being consumed after reduction (Híc et al., 2023). This process significantly inhibits both organic acid metabolism and carbohydrate degradation (Híc et al., 2023). The increase in SSC and SSC/TA, alongside the decrease in flesh firmness and TA, indicated continued fruit ripening during cold-storage (Cheng et al., 2021). Long-term cold storage of fruits might lead to a decrease in fruit quality, thereby reduced food value or even completely lost it (Mao et al., 2022). To a certain extent, the firmness, SSC, pH, sucrose and sorbitol can serve as an indicator of the relative maturity and quality of pears at the end of storage (Liu et al., 2019 & Mattheis et al., 2022). In this study, during the 0–4 M cold-storage, pears showed a trend of continued ripening, indicating that the continued ripening of pears during cold-storage was not long-lasting. After 6 M of cold-storage, pears exhibited increased TA, decreased SSC/TA and TSS/TA, and a decrease in SSC during the 7–14 d shelf-life, a profile contrary to the phenomenon of continued ripening, and pears with higher TSS/TA usually have better flavor (Xu et al., 2022). This change did not meet the customer requirements for pear fruit quality (Fig. 2). It may be speculated that pear fruit maturation ceases at this stage. Sensory evaluation shows that the shorter the cold-storage time is, the more juicy, sweet and fragrant the fruit (Predieri and Gatti, 2009). Consumer acceptance of pears has been shown to decrease after 23 weeks of cold-storage (Predieri and Gatti, 2009), suggesting that the cold-storage time of pear varieties tested should not exceed 6 months.

### 3.2 The relationship between quality traits of pears at different cold-storage periods

**Fig. 3:**
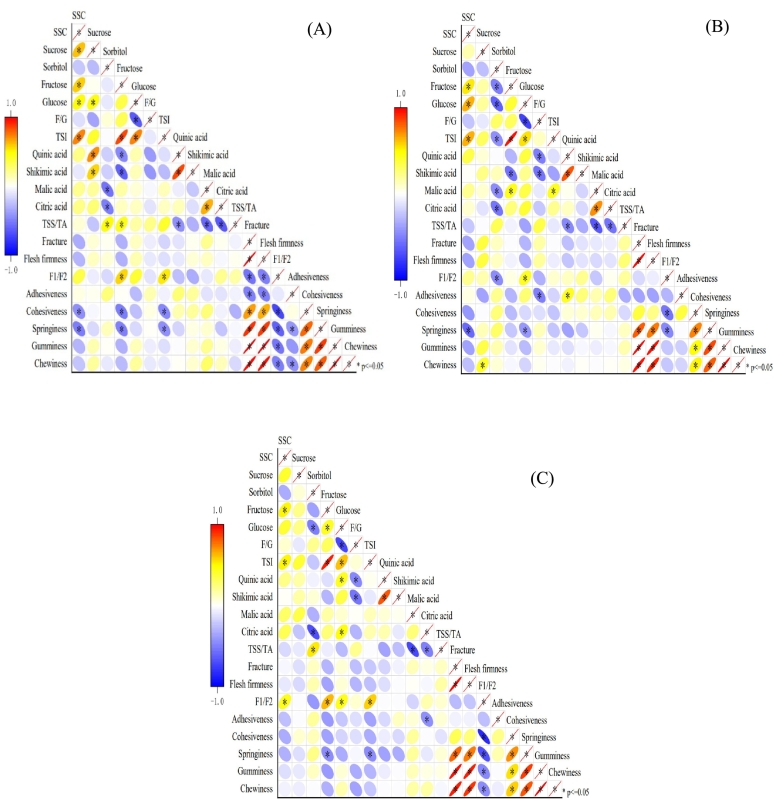
Pearson correlation analysis heatmap of pear quality traits at shelf-life after 2 (A), 4 (B), and 6 (C) months of cold-storage times.

The correlations among pear fruit quality traits varied with different cold-storage times. During the 0–14 d shelf-life after 2 M of cold-storage, SSC, fructose, TSI showed significant negative correlations with cohesiveness and springiness, while fructose and TSI were significantly positively correlated with F1/F2 (Fig. 3A). During the 0–14 d shelf-life after 4 M of cold-storage, SSC and glucose were significantly negatively correlated with springiness, whereas sorbitol and glucose were significantly correlated with F1/F2 (Fig. 3B). During the 0–14 d shelf-life after 6 M of cold-storage, SSC, fructose, glucose, TSI were significantly positively correlated with F1/F2, while fructose and TSI were significantly negatively correlated with springiness (Fig. 3C). After cold-storage, the sugar components of pears showed higher correlation with texture traits compared to organic acid components, particularly with F1/F2, cohesiveness and springiness. In pears without cold-storage treatment, flesh firmness was significantly positively correlated with sucrose and malic acid, and negatively correlated with glucose (Yin et al., 2025). After cold-storage, these significant correlations between flesh firmness and sugar or organic acid components were no longer observed (Fig. 3). Cold-storage might result in the differential modifications of gene expression of traits, leading to significant changes in the way their shared actions (pleiotropy) or their trait-specific actions (linkage-disequilibrium) affect fruit sensory characteristics. Notably, the overall variation in flesh firmness before and after cold-storage remained largely consistent, which further verified that cold-storage would cause sugar metabolism disorder (Wang et al., 2020).

**Fig. 4:**
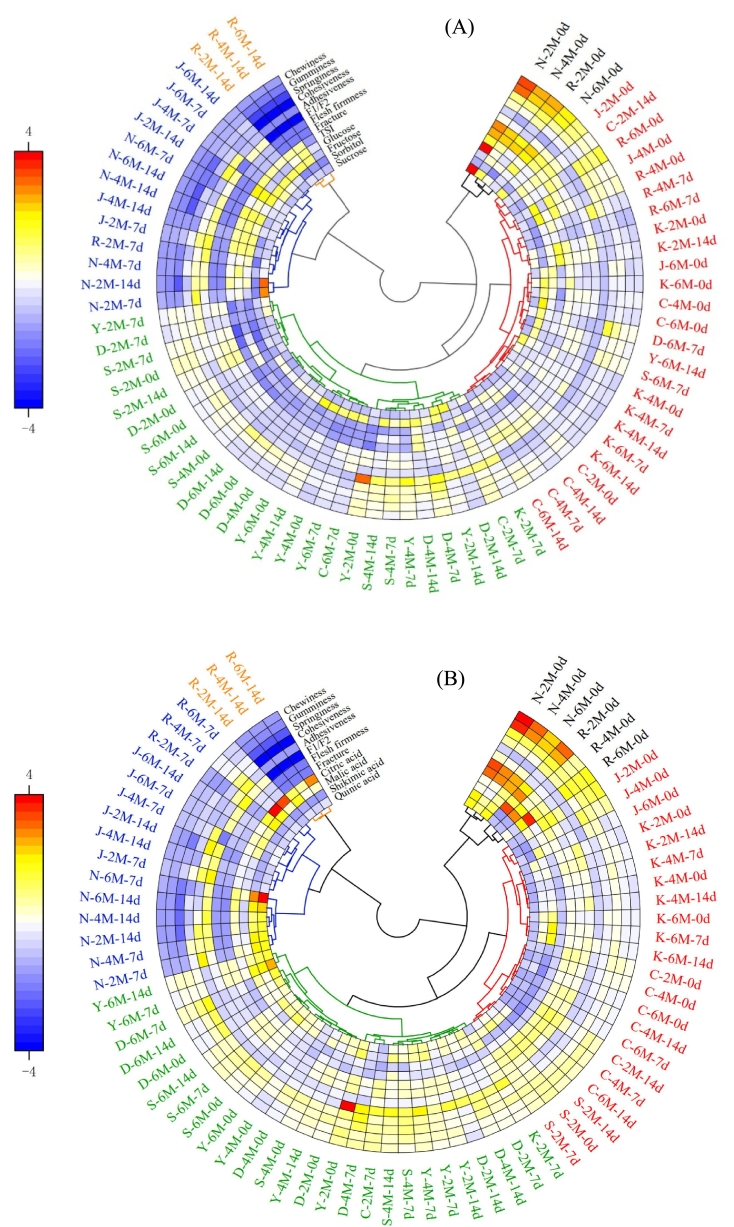
Cluster analysis heatmap of pears during shelf-life after different cold-storage times. Cluster analysis based on texture traits and sugar components (A), and on texture traits and organic acid components (B).

We adopted texture traits and sugar components for cluster analysis of pear fruits after different cold storage times and revealed that 72 samples fell into five distinct groups (Fig. 4A). Under our experimental conditions, Group I were Nanguoli with a shelf-life of 0 d after 2, 4 and 6 M of cold-storage. Group II included Jingbaili with a shelf-life of 0 d across all cold-storage times, Ruanerli with a shelf-life of 0–7 d after 4 and 6 M of cold-storage, and most Korla pears and Chili at a shelf-life of 0–14 d across all cold-storage times. Group III were most Dangshan Suli, Yali, and Shuihongxiao with a shelf-life of 0–14 d across all cold-storage times. Group IV included Nanguoli and Jingbaili with a shelf-life of 7–14 d across all cold-storage times. Group V were Ruanerli with a shelf-life of 14 d across all cold-storage times (Fig. 4A). The texture traits and sugar components of Chili and Korla pears were highly similar across cold-storage periods, as were those of Dangshan Suli, Yali and Shuihongxiao. Additionally, soft-flesh pears at 0 d of shelf-life showed high similarity in the texture traits and sugar components after different cold-storage times, which were similar to crispy-flesh pears. The texture traits and sugar components of Nanguoli and Jingbaili at the shelf-life of 7–14 d after different cold-storage times exhibited highly consistent profiles.

Based on texture traits and organic acid components, we performed cluster analysis of pear fruits subjected to different cold-storage times. The results revealed that the 72 samples were divided into five distinct groups (Fig. 4B). Under our experimental conditions, Group I included Nanguoli and Ruanerli with the shelf-life of 0 d across all cold-storage times. Group II contained Jingbaili with the shelf-life of 0 d across all cold-storage times, Shuihongxiao with the shelf-life of 0–14 d after 2 M cold-storage, and most Chili and Korla pears with the shelf-life of 0–14 d across all cold-storage times. Group III included Dangshan Suli and Yali with the shelf-life of 0–14 d across all cold-storage times, and Shuihongxiao with the shelf-life of 0–14 d after 4 and 6 M cold-storage. Group IV included Nanguoli and Jingbaili with the shelf-life of 7–14 d across all cold-storage times, and Ruanerli with the shelf-life of 7 d across all cold-storage times. Group V included Ruanerli with the shelf-life of 14 d across all cold-storage times (Fig. 4B). The texture traits and organic acid components of Korla pear and Chili were highly similar across cold-storage times, as were those of Dangshan Suli and Yali. The texture traits and organic acid components of Nanguoli and Ruanerli at the shelf-life of 0 d after different cold-storage times were highly similar, while Jingbaili at the shelf-life of 0 d after different cold-storage times showed greater similarity to Korla pear and Chili. According to previous studies, the genetic and phenotypic correlation between firmness and sugar was moderate and close, whereas the genetic and phenotypic correlation between firmness and organic acids was very low (Kouassi et al., 2009). For acidity, previous studies have illustrated a wide genetic variability between the genotypes for their acidity at harvest while they become more homogenous during storage (Kouassi et al., 2009). Although our Pearson correlation results indicated a stronger relationship between the sugar components and texture traits in pear (Fig. 3), the combination of texture traits and organic acid components more effectively classified the pear samples during the shelf-life following different cold-storage times. This might occur because changes in organic acids align with textural changes during cold storage, independent of functional interactions. Therefore, integrating texture traits with organic acid components provided a better evaluation of pear quality during the shelf-life after different cold-storage times.

### 3.3 Evaluation of pear quality traits after cold-storage

#### 3.3.1 Screening of differential traits during cold-storage

**Fig. 5:**
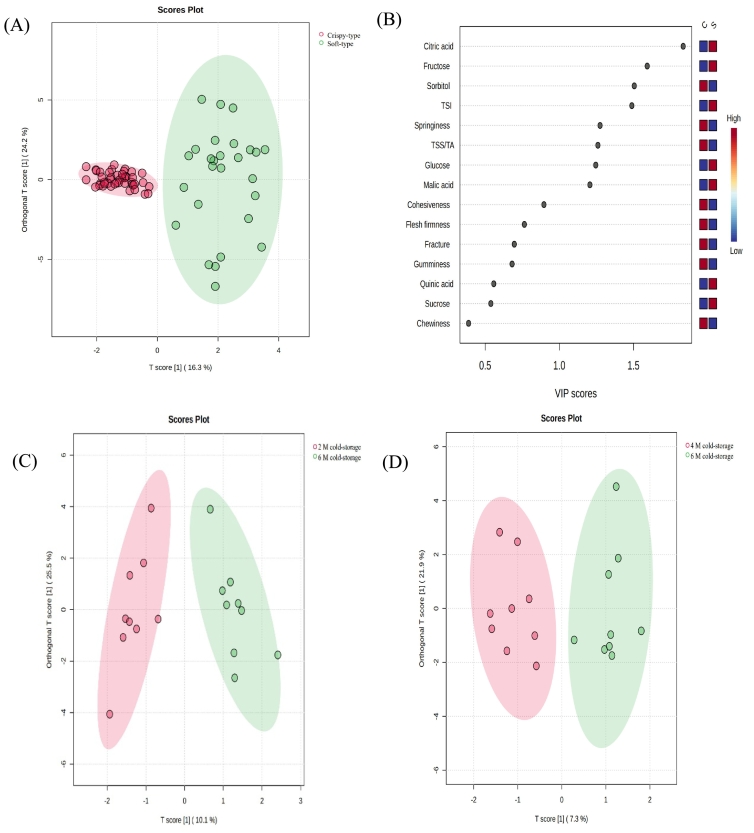
OPLS-DA heatmap of pears after different cold-storage times. OPLS-DA (A), and OPLS-DA VIP score (B) of soft-flesh pears and crispy-flesh pears after different cold-storage times. OPLS-DA of soft-flesh pears after 2 and 6 M of cold-storage (C). OPLS-DA of soft-flesh pears after 4 and 6 M of cold-storage (D).

OPLS-DA was conducted on the soft-flesh pears and crispy-flesh pears after different cold-storage times. The score plot showed a clear separation between soft-flesh pears and crispy-flesh pears (Fig. 5A). In cross validation (permutation, *n* = 1000), Q2 was 0.811 and R2Y was 0.871, indicating that the model had a good predictive ability. According to the OPLS-DA VIP score plot, the differential traits with VIP values greater than 1 were citric acid, fructose, sorbitol, TSI, springiness, TSS/TA, glucose and malic acid (Fig. 5B). Crispy-flesh pears have sufficiently strong flesh, allowing them to maintain firmness to a certain extent during storage (Híc et al., 2023). The soft-flesh pears selected in this study all belong to *Pyrus ussuriensis*, which undergo softening after harvest. The rate of fruit softening is a main factor determining postharvest deterioration (Brummell and Harpster, 2001). Softening and texture changes during storage are largely attributed to the breakdown of fruit cell wall (Brummell and Harpster, 2001). The swelling and softening of cell wall are evident in the form of soft and melted texture during maturation in fruits such as strawberries and avocados (Brummell and Harpster, 2001). But in fruit such as apple, which ripens to a crisp, fracturable texture, cell wall swelling is not observed (Brummell and Harpster, 2001). Our previous research revealed that, without cold-storage treatment, the differential traits in the OPLS-DA VIP analysis between soft-flesh pears and crispy-flesh pear in the OPLS-DA VIP analysis were sucrose, sorbitol, TSI, SSC, citric acid, malic acid and TSS/TA (Yin et al., 2025). It could be speculated that although cold-storage leads to changes in quality traits, the variation patterns of sorbitol, TSI, citric acid, malic acid and TSS/TA are closely associated with the texture type of pear fruit.

OPLS-DA was performed on the soft-flesh pears after different cold-storage times. The results showed that the soft-flesh pears cold-stored for 2 and 4 M were clearly separated from those cold-stored for 6 M, respectively (Fig. 5C & 5D). However, the soft-flesh pears cold-stored for 2 and 4 M were not effectively distinguished from each other (Fig. S1). In cross validation (permutation, n = 1000), the Q2 and R2Y of the soft-flesh pears cold-stored for 2 and 6 M model were 0.67 and 0.91, respectively, indicating that the model has a good predictive ability. The Q2 and R2Y of the soft-flesh pears cold-stored for 4 and 6 M model were 0.64 and 0.90, respectively, indicating that the model has a good predictive ability. Compared with samples cold-stored for 2 and 4 M, the quality traits of soft-flesh pears undergo significant changes after 6 M of cold-storage. According to the OPLS-DA VIP score plot, the differential traits with VIP values greater than 1 included adhesiveness, TSI, TSS/TA, sucrose, fructose, glucose, citric acid, sorbitol and F/G (Table. S4). In contrast, OPLS-DA applied to crispy- flesh pears cold-stored for different times revealed no clear separation among samples (Fig. S1). After cold-storage, soft-flesh pears underwent more pronounced quality changes than crispy-flesh pears. Low temperature regulation may induce transcription factors related to fruit softening and sugar accumulation (Dias et al., 2022). Soluble sugars have been found to alleviate the damage of cell membrane structure by inhibiting the transition of cell membrane from a gel to a liquid crystalline phase (Wang et al., 2020). Sucrose, in particular, helps regulate osmotic potential, maintain cellular turgor, preserve water content, stabilize the phospholipid bilayer structure, and reduce the damage of low-temperature to biofilm (Yang et al., 2024). It also exhibits stronger membrane protection capacity than reducing sugar (Wang et al., 2013). The OPLS-DA result showed that most differential traits were associated with sugar and organic acid components, supporting the potential use of individual sugars and organic acids as reliable candidates for evaluating fruit quality.

#### 3.3.2 Evaluation of pears after different cold-storage times

The PCA scores based on texture traits and sugar and acid components of pears after different cold-storage times were presented in Table 3. The analysis yielded 6 eigenvalues greater than 1. The first principal component accounted for 28.32 % of the total variation, and the second principal component, 17.74 %. The fracture, flesh firmness, springiness, gumminess and chewiness had significant loads on the first principal component, representing texture-related traits, while sucrose, sorbitol and F/G had significant loads on the second principal component. Combined with cluster analysis (Fig. 4B), these results indicated that texture traits were also an indispensable component in fruit classification and quality evaluation.

**Table 3:** PCA score of pear varieties during shelf-life after different cold-storage times.

Nanguoli									
Cold-storage time	2 M			4 M			6 M		
Score	0.371			0.053			−0.090		
Shelf-life/d	0	7	14	0	7	14	0	7	14
Score	1.78	−0.278	−0.389	1.129	−0.448	−0.524	0.856	−0.623	−0.502
Jingbaili									
Cold-storage time	2 M			4 M			6 M		
Score	−0.017			−0.111			−0.318		
Shelf-life/d	0	7	14	0	7	14	0	7	14
Score	0.526	−0.278	−0.3	0.291	−0.296	−0.327	−0.102	−0.412	−0.441
Ruanerli									
Cold-storage time	2 M			4 M			6 M		
Score	0.052			0			−0.036		
Shelf-life/d	0	7	14	0	7	14	0	7	14
Score	0.814	−0.657	–	0.252	−0.253	–	0.373	−0.482	–
Korla pear									
Cold-storage time	2 M			4 M			6 M		
Score	0.121			0.039			−0.026		
Shelf-life/d	0	7	14	0	7	14	0	7	14
Score	0.129	−0.018	0.252	0.034	0.113	−0.032	0.101	−0.087	−0.092
Chili									
Cold-storage time	2 M			4 M			6 M		
Score	0.159			0.125			0.056		
Shelf-life/d	0	7	14	0	7	14	0	7	14
Score	−0.084	0.183	0.377	0.107	0.259	0.010	0.100	−0.136	0.204
Dangshan Suli									
Cold-storage time	2 M			4 M			6 M		
Score	0.010			0.146			−0.062		
Shelf-life/d	0	7	14	0	7	14	0	7	14
Score	0.020	−0.120	0.132	−0.114	0.282	0.269	−0.214	0.198	−0.170
Yali									
Cold-storage time	2 M			4 M			6 M		
Score	0.000			−0.145			−0.116		
Shelf-life/d	0	7	14	0	7	14	0	7	14
Score	−0.067	−0.131	0.199	−0.379	−0.016	−0.039	−0.135	−0.232	0.018
Shuihongxiao									
Cold-storage time	2 M			4 M			6 M		
Score	−0.053			−0.032			−0.125		
Shelf-life/d	0	7	14	0	7	14	0	7	14
Score	−0.026	−0.185	0.053	−0.181	0.064	0.020	−0.219	0.033	−0.190

Note: – represents the data was empty.

Soft-flesh pears achieved the highest overall quality score after 2 M of cold-storage. Furthermore, at each of cold-storage period, the highest quality scores were consistently observed at 0 d of shelf-life. Nanguoli, Jingbaili and Ruanerli are mid-maturity varieties. Based on our previous research, we speculated that the optimal harvest time for these varieties was estimated to be around 146 DAFB (Yin et al., 2025). Since pear fruits continue to mature during cold storage, the quality scores of Nanguoli, Jingbaili and Ruanerli gradually declined as cold-storage time was extended (Table 3). This trend indicated that the maturity of Nanguoli, Jingbaili, and Ruanerli harvested at 146 DAFB was quite good. Therefore, the suitable cold-storage time for Nanguoli, Jingbaili and Ruanerli harvested at 146 DAFB was 2 months, with a suitable shelf-life of 0–7 days.

Korla pears achieved the highest overall quality score after 2 M of cold-storage. Specifically, the highest scores were observed at the shelf-life of 14 d after 2 M of cold-storage, at the shelf-life of 7 d after 4 M of cold-storage, and at the shelf-life of 0 d after 6 M of cold-storage, respectively (Table 3). Chili and Yali also achieved the highest overall quality score after the 2 M of cold-storage, with peak score recorded at the shelf-life of 14 d after both 2 and 6 M cold-storage, and at the shelf-life of 7 d after 4 M cold-storage, respectively (Table 3). Although Korla pear, Chili and Yali are late-maturity varieties, our previous studies speculated that the suitable harvest time was estimated to be around 146 DAFB (Yin et al., 2025). The quality scores of Korla pear, Chili and Yali gradually decreased as cold-storage time increased (Table 3). This trend indicated that Korla pear, Chili and Yali harvested at 146 DAFB had reached an appropriate maturity level.

Dangshan Suli and Shuihongxiao achieved the highest overall quality score after 4 M of cold-storage. Specifically, the highest scores were observed at the shelf-life of 14 d after 2 M of cold-storage, and at the shelf-life of 7 d after both 4 and 6 M of cold-storage, respectively (Table 3). Dangshan Suli and Shuihongxiao are also late-maturity varieties. We speculated that fruits harvested at 146 DAFB may not yet have reached their optimal harvest maturity in previous studies (Yin et al., 2025). This was further supported by the tendency in quality scores, which initially increased and then decreased with prolonged cold-storage (Table 3). This result indicated that compared with other pear varieties, Dangshan Suli and Shuihongxiao harvested at 146 DAFB were slightly less mature. They might require either a certain amount of cold-storage or a later harvest date to achieve optimal quality. Our findings demonstrate that integrating texture traits with variation in sugar and organic acid content help to infer the suitable cold-storage period and optimal shelf-life for different pear varieties. The suitable cold-storage length appears closely related to fruit maturity at harvest. In the future, fruit maturity may be used as a key indicator to further refine the inference of the suitable cold-storage period and shelf-life for individual pear varieties.

## 4 Conclusion

The results indicated that the tested pears exhibited a decrease in SSC, sucrose, and organic acid components in response to cold storage, while their fructose, sorbitol, and glucose content increased. However, prolonged cold-storage resulted in a decrease in fruit quality, suggesting that the cold-storage time of tested pears should not exceed more than 6 months. After cold-storage, no significant difference was observed in sucrose content between high-sucrose soft-flesh pears and low-sucrose crispy-flesh pears, and that eventually disappeared with extended storage. The sharp decline of sucrose in soft-flesh pears caused by cold-storage contributes to the cold-induced sweetening, and it may be associated with earlier softening process. Sugar components were significantly correlated with F1/F2, cohesiveness and springiness during cold-storage. Based on PCA, Nanguoli, Jingbaili, Ruanerli, Korla pear, Chili and Yali achieved the highest fruit quality score after 2 months of cold-storage, while Dangshan Suli and Shuihongxiao after 4 months. Texture traits, sugar and organic acid components can serve as key evaluation indicators to inform decisions on both appropriate cold-storage length and shelf-life, providing theoretical references for the construction of postharvest cold-storage strategies for pears.

## CRediT authorship contribution statement

**Chen Yin:** Writing – original draft, Methodology, Formal analysis, Data curation. **Luming Tian:** Writing – review & editing, Supervision, Funding acquisition, Conceptualization. **Hongliang Huo:** Writing – review & editing, Resources. **Jing Li:** Methodology. **Xingguang Dong:** Writing – review & editing. **Ying Zhang:** Writing – review & editing. **Dan Qi:** Data curation. **Jiayu Xu:** Visualization. **Chao Liu:** Formal analysis. **Yufen Cao:** Writing – review & editing.

## Declaration of competing interest

The authors declare that they have no known competing financial interests or personal relationships that could have appeared to influence the work reported in this paper.

## Data Availability

Data will be made available on request.

## Appendix A Supplementary data

Supplementary material Image 1
