# Supplementary material for: Evaluating fruit quality of pear varieties with different textures after diverse cold-storage times
Source: Food Chem X. 2025 Dec 1;32:103357. doi: 10.1016/j.fochx.2025.103357 (PMC12723134; doi:10.1016/j.fochx.2025.103357)
Supplement: Supplementary file 1 — Supplementary material [file mmc1.docx]

Supplementary Materials

Table S1 Calibration slope of standard individual sugars and organic acids.

| components | R2 | calibration slope |
| --- | --- | --- |
| sucrose | 0.999016 | 1013.57*X |
| sorbitol | 0.991821 | 6.42247*X2+1324.99*X-855.179 |
| fructose | 0.995876 | -0.987461*X2+729.348*X-256.52 |
| glucose | 0.999918 | 12.3067*X2-1.28871*X |
| quinic acid | 0.998655 | 64.9581*X-12.1241 |
| shikimic acid | 0.999446 | 53.5912*X+13.067 |
| malic acid | 0.993215 | 87.0462*X-225.613 |
| succinic acid | 0.998689 | 43.1208*X-1.19056 |
| citric acid | 0.993469 | 109.152*X-402.783 |
| fumaric acid | 0.999338 | 27.6996*X-0.343545 |

Table S2

Variation of sugar and organic acid components in pear fruits during shelf life of 0-14 d after different cold storage times.

|  | Nanguoli | Jingbaili | Ruanerli | Korla pear | Chili | Dangshan Suli | Yali | Shuihongxiao |
| --- | --- | --- | --- | --- | --- | --- | --- | --- |
| SSC | | | | | | | | |
| Cold storage 0 M-0 d | 12.83 | 12.9 | 12.82 | 13.09 | 13.58 | 11.7 | 13.02 | 10.51 |
| Cold storage 0 M-7 d | 12.79 | 14.27 | 13.83 | 12.69 | 13.07 | 11.27 | 11.55 | 9.5 |
| Cold storage 0 M-14 d | 16.63 | 16.53 | 15.88 | 13.55 | 13.67 | 12.69 | 11.43 | 12 |
| Cold storage 2 M-0 d | 13.69 | 14.08 | 12.04 | 11.24 | 11.17 | 11.97 | 10.43 | 9.52 |
| Cold storage 2 M-7 d | 14.84 | 13.43 | 13.25 | 11.53 | 12.05 | 12.94 | 11.41 | 10.46 |
| Table S2 (continue) | | | | | | | | |
| Cold storage 2 M-14 d | 14.14 | 14.22 | - | 13.53 | 12.81 | 13.25 | 12.66 | 11.15 |
| Cold storage 4 M-0 d | 14.48 | 13.51 | 13.9 | 12.57 | 12.39 | 13.87 | 11.86 | 10.98 |
| Cold storage 4 M-7 d | 14.65 | 14.89 | 12.54 | 13.36 | 12.78 | 13.1 | 12.06 | 11.24 |
| Cold storage 4 M-14 d | 16.23 | 16.42 | - | 12.44 | 12.02 | 14.03 | 13.57 | 13.37 |
| Cold storage 6 M-0 d | 16.1 | 15.06 | 14.97 | 13.74 | 13.35 | 14.04 | 12.99 | 12.32 |
| Cold storage 6 M-7 d | 16.89 | 14.7 | 13.45 | 13.81 | 13.9 | 13.38 | 13.38 | 12.23 |
| Cold storage 6 M-14 d | 14.18 | 13.85 | - | 13.73 | 12.45 | 11.79 | 11.29 | 9.96 |
| Sucrose | | | | | | | | |
| Cold storage 0 M-0 d | 8.8871 | 9.1911 | 5.4875 | 2.441 | 1.2892 | 1.603 | 2.1812 | 0.6243 |
| Cold storage 0 M-7 d | 11.314 | 2.818 | 1.8688 | 2.5903 | 1.7062 | 1.3446 | 1.4941 | 0.7024 |
| Cold storage 0 M-14 d | 8.3705 | 0.8204 | 1.206 | 1.7206 | 1.2945 | 2.3372 | 2.4544 | 1.0813 |
| Cold storage 2 M-0 d | 4.8725 | 2.2908 | 1.4925 | 1.4582 | 0.3674 | 1.0515 | 0.9864 | 0.6506 |
| Cold storage 2 M-7 d | 3.8614 | 0.7229 | 1.2314 | 1.4967 | 0.9377 | 1.5922 | 1.4948 | 0.6235 |
| Cold storage 2 M-14 d | 4.0317 | 0.9438 | 0.9469 | 1.3261 | 0.9926 | 2.5584 | 2.4317 | 0.685 |
| Cold storage 4 M-0 d | 2.493 | 0.8082 | 0.8687 | 2.3336 | 0.8803 | 0.8759 | 0.8222 | 0.4538 |
| Cold storage 4 M-7 d | 1.1029 | 0.5121 | 0.7577 | 1.4068 | 0.6819 | 1.0267 | 0.7549 | 0.5384 |
| Cold storage 4 M-14 d | 1.0134 | 0.6486 | 0.7146 | 1.027 | 0.5759 | 1.031 | 0.549 | 0.4527 |
| Cold storage 6 M-0 d | 0.8366 | 0.6607 | 0.5628 | 1.4234 | 0.4697 | 0.7363 | 0.5439 | 0.4154 |
| Cold storage 6 M-7 d | 0.929 | 0.6099 | 0.4436 | 1.2477 | 0.5187 | 1.2023 | 0.5643 | 0.3831 |
| Cold storage 6 M-14 d | 0.8533 | 0.4062 | 0.3826 | 0.5662 | 0.468 | 0.8732 | 0.2674 | 0.3319 |
| Sorbitol | | | | | | | | |
| Cold storage 0 M-0 d | 1.9573 | 2.9214 | 1.2143 | 3.064 | 3.82 | 2.8422 | 3.8677 | 2.8136 |
| Cold storage 0 M-7 d | 1.2756 | 2.238 | 0.9798 | 2.7871 | 2.8299 | 2.3112 | 3.3805 | 2.4625 |
| Cold storage 0 M-14 d | 1.2872 | 1.8264 | 0.7134 | 2.0263 | 2.2989 | 2.4946 | 3.8732 | 2.8851 |
| Cold storage 2 M-0 d | 2.2382 | 2.5048 | 1.1953 | 2.9563 | 3.581 | 2.4234 | 3.5292 | 2.6276 |
| Table S2 (continue) | | | | | | | | |
| Cold storage 2 M-7 d | 1.2549 | 2.8829 | 1.0238 | 2.4856 | 3.6895 | 2.8942 | 3.1688 | 2.5218 |
| Cold storage 2 M-14 d | 1.5086 | 4.1334 | 1.0688 | 2.3836 | 3.6668 | 3.4198 | 6.3588 | 3.6777 |
| Cold storage 4 M-0 d | 3.0398 | 4.014 | 2.1977 | 4.7342 | 6.5012 | 4.6068 | 6.1013 | 4.27 |
| Cold storage 4 M-7 d | 2.6085 | 5.7935 | 2.0018 | 4.023 | 5.2169 | 5.5291 | 6.3167 | 5.2814 |
| Cold storage 4 M-14 d | 3.7319 | 1.9291 | 1.9291 | 3.7863 | 4.736 | 6.9771 | 7.3655 | 6.6324 |
| Cold storage 6 M-0 d | 3.6754 | 5.2217 | 2.1049 | 6.8271 | 8.195 | 4.7521 | 7.819 | 4.9957 |
| Cold storage 6 M-7 d | 3.6011 | 4.5987 | 1.7389 | 4.9165 | 6.2439 | 6.5158 | 6.8383 | 5.7101 |
| Cold storage 6 M-14 d | 2.915 | 3.893 | 1.141 | 3.3875 | 4.6219 | 4.7827 | 6.0859 | 4.9173 |
| Fructose | | | | | | | | |
| Cold storage 0 M-0 d | 5.1056 | 6.0675 | 6.6942 | 8.2735 | 8.2993 | 6.8232 | 5.0906 | 5.6032 |
| Cold storage 0 M-7 d | 5.0607 | 10.0121 | 8.309 | 7.6626 | 8.0115 | 7.3326 | 4.9945 | 4.8168 |
| Cold storage 0 M-14 d | 6.1926 | 10.8668 | 8.575 | 8.1682 | 9.09 | 8.9973 | 6.5593 | 5.793 |
| Cold storage 2 M-0 d | 7.6127 | 8.7459 | 9.5733 | 9.3701 | 9.52 | 6.3515 | 5.5881 | 4.7693 |
| Cold storage 2 M-7 d | 7.8189 | 11.2046 | 9.7677 | 8.9227 | 9.8401 | 6.3242 | 5.4508 | 4.8916 |
| Cold storage 2 M-14 d | 9.8203 | 12.3775 | 11.7661 | 9.2635 | 10.5331 | 8.3056 | 6.7983 | 5.8114 |
| Cold storage 4 M-0 d | 10.0029 | 12.5812 | 12.7163 | 8.9941 | 9.5032 | 7.4904 | 6.5886 | 6.0548 |
| Cold storage 4 M-7 d | 8.8746 | 11.7191 | 9.0696 | 8.5724 | 8.6406 | 8.1425 | 6.6575 | 6.7627 |
| Cold storage 4 M-14 d | 9.8563 | 10.9473 | 10.9473 | 9.5629 | 9.9372 | 9.3181 | 7.0956 | 6.2837 |
| Cold storage 6 M-0 d | 8.666 | 10.2593 | 9.4221 | 9.8675 | 9.1097 | 7.4194 | 5.9088 | 6.5097 |
| Cold storage 6 M-7 d | 8.7945 | 11.5768 | 8.7878 | 8.4858 | 6.8178 | 8.6062 | 5.9796 | 7.7603 |
| Cold storage 6 M-14 d | 11.5004 | 11.5015 | 7.4276 | 8.6431 | 8.0059 | 6.9953 | 8.4226 | 5.9161 |
| Glucose | | | | | | | | |
| Cold storage 0 M-0 d | 1.8032 | 1.0718 | 2.6349 | 8.0425 | 2.5269 | 3.1165 | 1.9891 | 3.7495 |
| Cold storage 0 M-7 d | 2.9813 | 7.0477 | 10.686 | 7.4194 | 2.5268 | 3.3523 | 4.0059 | 3.94 |
| Cold storage 0 M-14 d | 6.4072 | 6.2633 | 9.3982 | 7.729 | 3.0727 | 5.7013 | 1.9348 | 5.4464 |
| Table S2 (continue) | | | | | | | | |
| Cold storage 2 M-0 d | 14.043 | 5.6835 | 5.4878 | 9.0649 | 2.9646 | 6.3727 | 3.3061 | 5.6323 |
| Cold storage 2 M-7 d | 6.2767 | 8.3745 | 6.2748 | 4.5247 | 3.6945 | 3.8457 | 3.6834 | 3.8802 |
| Cold storage 2 M-14 d | 6.404 | 9.5413 | 6.393 | 9.1801 | 6.4383 | 7.127 | 6.193 | 6.4285 |
| Cold storage 4 M-0 d | 6.5813 | 5.1085 | 6.5682 | 4.8 | 3.4262 | 4.0731 | 2.5179 | 4.3489 |
| Cold storage 4 M-7 d | 8.0207 | 4.9217 | 7.109 | 6.1068 | 4.4084 | 5.703 | 4.1441 | 5.7799 |
| Cold storage 4 M-14 d | 8.3078 | 8.0564 | 8.0564 | 5.9639 | 4.0675 | 7.3355 | 3.6358 | 5.8581 |
| Cold storage 6 M-0 d | 8.5861 | 4.0238 | 6.1441 | 5.5349 | 4.1769 | 3.1818 | 4.5109 | 3.1021 |
| Cold storage 6 M-7 d | 7.0617 | 4.2584 | 6.2061 | 4.4564 | 3.0708 | 5.4075 | 3.051 | 5.0745 |
| Cold storage 6 M-14 d | 8.7577 | 4.1095 | 5.53 | 5.867 | 5.3029 | 3.995 | 6.0367 | 4.2968 |
| F/G | | | | | | | | |
| Cold storage 0 M-0 d | 2.83 | 5.66 | 2.54 | 1.03 | 3.28 | 2.19 | 2.56 | 1.49 |
| Cold storage 0 M-7 d | 1.7 | 1.42 | 0.78 | 1.03 | 3.17 | 2.19 | 1.25 | 1.22 |
| Cold storage 0 M-14 d | 0.97 | 1.73 | 0.91 | 1.06 | 2.96 | 1.58 | 3.39 | 1.06 |
| Cold storage 2 M-0 d | 0.54 | 1.54 | 1.74 | 1.03 | 3.21 | 1 | 1.69 | 0.85 |
| Cold storage 2 M-7 d | 1.25 | 1.34 | 1.56 | 1.97 | 2.66 | 1.64 | 1.48 | 1.26 |
| Cold storage 2 M-14 d | 1.53 | 1.3 | 1.84 | 1.01 | 1.64 | 1.17 | 1.1 | 0.9 |
| Cold storage 4 M-0 d | 1.52 | 2.46 | 1.94 | 1.87 | 2.77 | 1.84 | 2.62 | 1.39 |
| Cold storage 4 M-7 d | 1.11 | 2.38 | 1.28 | 1.4 | 1.96 | 1.43 | 1.61 | 1.17 |
| Cold storage 4 M-14 d | 1.19 | 1.36 | 1.36 | 1.6 | 2.44 | 1.27 | 1.95 | 1.07 |
| Cold storage 6 M-0 d | 1.01 | 2.55 | 1.53 | 1.78 | 2.18 | 2.33 | 1.31 | 2.1 |
| Cold storage 6 M-7 d | 1.25 | 2.72 | 1.42 | 1.9 | 2.22 | 1.59 | 1.96 | 1.53 |
| Cold storage 6 M-14 d | 1.31 | 2.8 | 1.34 | 1.47 | 1.51 | 1.75 | 1.4 | 1.38 |
| TSI | | | | | | | | |
| Cold storage 0 M-0 d | 20.09 | 22.06 | 19.57 | 24.38 | 19.59 | 17.25 | 14.65 | 14.65 |
| Cold storage 0 M-7 d | 22.92 | 26.47 | 24.6 | 22.85 | 18.92 | 17.71 | 15.02 | 13.32 |
| Table S2 (continue) | | | | | | | | |
| Cold storage 0 M-14 d | 24.48 | 25.09 | 23.26 | 22.62 | 20.43 | 23.41 | 17.38 | 16.75 |
| Cold storage 2 M-0 d | 29.69 | 22.92 | 22.6 | 25.96 | 20.92 | 18.08 | 15.08 | 14.56 |
| Cold storage 2 M-7 d | 22.61 | 27.78 | 23.16 | 21.55 | 22.65 | 16.96 | 15.42 | 13.36 |
| Cold storage 2 M-14 d | 26.43 | 31.62 | 26.39 | 25.39 | 25.93 | 24.08 | 22.45 | 17.59 |
| Cold storage 4 M-0 d | 26.26 | 28.44 | 28.73 | 24.06 | 23.51 | 19.43 | 17.57 | 16.57 |
| Cold storage 4 M-7 d | 23.77 | 27.6 | 22.71 | 22.97 | 21.81 | 22.46 | 18.97 | 19.54 |
| Cold storage 4 M-14 d | 26.24 | 26.46 | 26.52 | 24.03 | 23.36 | 26.56 | 19.76 | 19.51 |
| Cold storage 6 M-0 d | 24.21 | 24.25 | 22.45 | 26.45 | 24.01 | 18.59 | 18.66 | 16.81 |
| Cold storage 6 M-7 d | 23.34 | 26.24 | 21.08 | 21.97 | 18.16 | 23.8 | 17.12 | 20.81 |
| Cold storage 6 M-14 d | 28.72 | 25.38 | 17.84 | 21.69 | 20.83 | 18.63 | 22.76 | 16.56 |
| Quinic acid | | | | | | | | |
| Cold storage 0 M-0 d | 0.591 | 0.166 | 0.32 | 0.125 | 0.256 | 0.502 | 0.481 | 0.341 |
| Cold storage 0 M-7 d | 0.362 | 0.175 | 0.214 | 0.147 | 0.216 | 0.324 | 0.339 | 0.181 |
| Cold storage 0 M-14 d | 0.443 | 0.16 | 0.277 | 0.182 | 0.149 | 0.272 | 0.369 | 0.247 |
| Cold storage 2 M-0 d | 0.562 | 0.14 | 0.128 | 0.144 | 0.151 | 0.358 | 0.377 | 0.312 |
| Cold storage 2 M-7 d | 0.592 | 0.208 | 0.206 | 0.152 | 0.142 | 0.404 | 0.406 | 0.249 |
| Cold storage 2 M-14 d | 0.517 | 0.172 | 0.23 | 0.123 | 0.088 | 0.34 | 0.326 | 0.253 |
| Cold storage 4 M-0 d | 0.433 | 0.088 | 0.148 | 0.104 | 0.083 | 0.226 | 0.286 | 0.254 |
| Cold storage 4 M-7 d | 0.599 | 0.241 | 0.224 | 0.0958 | 0.0964 | 0.251 | 0.417 | 0.287 |
| Cold storage 4 M-14 d | 0.544 | 0.169 | 0.163 | 0.105 | 0.092 | 0.3005 | 0.341 | 0.313 |
| Cold storage 6 M-0 d | 0.572 | 0.157 | 0.301 | 0.175 | 0.154 | 0.333 | 0.374 | 0.349 |
| Cold storage 6 M-7 d | 0.932 | 0.264 | 0.14 | 0.14 | 0.135 | 0.533 | 0.7005 | 0.352 |
| Cold storage 6 M-14 d | 0.675 | 0.156 | 0.23 | 0.146 | 0.226 | 0.426 | 0.513 | 0.328 |
| Shikimic acid | | | | | | | | |
| Cold storage 0 M-0 d | 0.177 | 0.035 | 0.066 | 0.1 | 0.112 | 0.146 | 0.14 | 0.152 |
| Table S2 (continue) | | | | | | | | |
| Cold storage 0 M-7 d | 0.104 | 0.036 | 0.072 | 0.115 | 0.085 | 0.099 | 0.109 | 0.084 |
| Cold storage 0 M-14 d | 0.122 | 0.034 | 0.055 | 0.135 | 0.056 | 0.098 | 0.095 | 0.124 |
| Cold storage 2 M-0 d | 0.176 | 0.043 | 0.021 | 0.106 | 0.058 | 0.102 | 0.139 | 0.127 |
| Cold storage 2 M-7 d | 0.181 | 0.04 | 0.032 | 0.1 | 0.05 | 0.108 | 0.128 | 0.103 |
| Cold storage 2 M-14 d | 0.139 | 0.032 | 0.033 | 0.083 | 0.025 | 0.105 | 0.076 | 0.102 |
| Cold storage 4 M-0 d | 0.121 | 0.02 | 0.025 | 0.081 | 0.044 | 0.062 | 0.101 | 0.102 |
| Cold storage 4 M-7 d | 0.179 | 0.0283 | 0.0372 | 0.066 | 0.0317 | 0.065 | 0.122 | 0.106 |
| Cold storage 4 M-14 d | 0.135 | 0.027 | 0.022 | 0.0746 | 0.033 | 0.086 | 0.085 | 0.128 |
| Cold storage 6 M-0 d | 0.145 | 0.0216 | 0.05004 | 0.107 | 0.065 | 0.0951 | 0.1004 | 0.144 |
| Cold storage 6 M-7 d | 0.223 | 0.054 | 0.0155 | 0.0966 | 0.0435 | 0.09 | 0.184 | 0.122 |
| Cold storage 6 M-14 d | 0.175 | 0.025 | 0.0205 | 0.104 | 0.089 | 0.095 | 0.147 | 0.128 |
| Malic acid | | | | | | | | |
| Cold storage 0 M-0 d | 2.655 | 2.712 | 5.501 | 2.397 | 2.135 | 2.019 | 3.034 | 1.063 |
| Cold storage 0 M-7 d | 1.919 | 2.411 | 5.01 | 2.833 | 1.963 | 1.35 | 2.419 | 0.468 |
| Cold storage 0 M-14 d | 2.146 | 2.225 | 5.261 | 3.133 | 1.47 | 1.415 | 2.101 | 1.067 |
| Cold storage 2 M-0 d | 2.087 | 2.412 | 5.013 | 1.723 | 0.943 | 1.618 | 1.795 | 1.153 |
| Cold storage 2 M-7 d | 2.167 | 1.593 | 3.758 | 2.465 | 0.817 | 1.784 | 1.83 | 0.964 |
| Cold storage 2 M-14 d | 1.279 | 1.453 | 3.45 | 1.991 | 0.679 | 1.647 | 1.55 | 0.846 |
| Cold storage 4 M-0 d | 1.796 | 1.494 | 4.581 | 1.92 | 0.642 | 1.274 | 1.672 | 0.958 |
| Cold storage 4 M-7 d | 1.918 | 2.613 | 3.609 | 1.541 | 0.401 | 1.208 | 1.906 | 1.099 |
| Cold storage 4 M-14 d | 1.306 | 1.663 | 1.961 | 2.123 | 0.875 | 1.868 | 1.64 | 1.332 |
| Cold storage 6 M-0 d | 2.559 | 2.681 | 3.9 | 2.833 | 0.41 | 2.133 | 1.751 | 1.989 |
| Cold storage 6 M-7 d | 2.271 | 2.761 | 2.186 | 3.735 | 0.654 | 2.664 | 3.424 | 1.425 |
| Cold storage 6 M-14 d | 1.396 | 2.447 | 2.279 | 3.414 | 0.598 | 2.093 | 2.481 | 1.775 |
| Citric acid | | | | | | | | |
| Table S2 (continue) | | | | | | | | |
| Cold storage 0 M-0 d | 2.982 | 1.757 | 5.387 | 0.185 | 0.771 | 0.15 | 2.13 | 2.176 |
| Cold storage 0 M-7 d | 2.331 | 2.316 | 3.725 | 0.28 | 1.182 | 0.128 | 1.738 | 1.763 |
| Cold storage 0 M-14 d | 3.168 | 2.181 | 6.216 | 0.199 | 0.617 | 0.153 | 2.175 | 1.836 |
| Cold storage 2 M-0 d | 2.057 | 1.632 | 3.992 | 0.231 | 0.554 | 0.126 | 0.672 | 1.587 |
| Cold storage 2 M-7 d | 2.347 | 1.246 | 4.564 | 0.199 | 0.491 | 0.147 | 1.043 | 1.695 |
| Cold storage 2 M-14 d | 2.474 | 1.209 | 4.323 | 0.292 | 0.621 | 0.149 | 1.455 | 1.561 |
| Cold storage 4 M-0 d | 2.119 | 1.805 | 4.98 | 0.128 | 0.344 | 0.105 | 0.807 | 1.231 |
| Cold storage 4 M-7 d | 1.699 | 1.971 | 9.289 | 0.13 | 0.208 | 0.109 | 0.763 | 1.629 |
| Cold storage 4 M-14 d | 2.284 | 1.501 | 3.107 | 0.19 | 0.35 | 0.113 | 0.868 | 2.295 |
| Cold storage 6 M-0 d | 3.812 | 1.904 | 8.609 | 0.134 | 0.365 | 0.086 | 1.094 | 2.122 |
| Cold storage 6 M-7 d | 3.832 | 2.276 | 8.391 | 0.273 | 0.31 | 0.088 | 1.792 | 2.138 |
| Cold storage 6 M-14 d | 3.81 | 2.043 | 7.436 | 0.209 | 0.265 | 0.111 | 1.446 | 1.865 |
| TSS/TA | | | | | | | | |
| Cold storage 0 M-0 d | 27.69 | 41.22 | 14.22 | 77.65 | 48.75 | 50.99 | 22.68 | 34.29 |
| Cold storage 0 M-7 d | 43.71 | 44.78 | 24.21 | 60.53 | 43.68 | 75.47 | 30.11 | 47.68 |
| Cold storage 0 M-14 d | 37.86 | 43 | 16.84 | 53.81 | 68.82 | 100.67 | 31.27 | 46.51 |
| Cold storage 2 M-0 d | 58.95 | 45.46 | 19.4 | 103.86 | 96.08 | 73.64 | 45 | 43.02 |
| Cold storage 2 M-7 d | 36.31 | 75.02 | 21.38 | 59.69 | 121.07 | 60.08 | 40.47 | 39.6 |
| Cold storage 2 M-14 d | 49.34 | 94.08 | 25.09 | 88.96 | 153.4 | 95.58 | 63.87 | 60.14 |
| Cold storage 4 M-0 d | 49.49 | 66.01 | 22.97 | 93.54 | 182.97 | 102.1 | 55.85 | 59.33 |
| Cold storage 4 M-7 d | 46.84 | 47.32 | 14.39 | 109.89 | 256.08 | 125.15 | 55.67 | 58.85 |
| Cold storage 4 M-14 d | 53.65 | 64.23 | 41.24 | 81.69 | 143.11 | 104.05 | 63.65 | 47.25 |
| Cold storage 6 M-0 d | 30.69 | 42.37 | 14.18 | 72.77 | 221.72 | 60.72 | 56.57 | 32.65 |
| Cold storage 6 M-7 d | 28.09 | 39.25 | 16.01 | 45.07 | 146.05 | 64.29 | 26.93 | 46.86 |
| Cold storage 6 M-14 d | 39.65 | 42.63 | 14.52 | 47.7 | 155.93 | 60.99 | 45.34 | 37.71 |

Table S3

Variation of texture traits in pear fruits during shelf life of 0-14 d after different cold storage times.

|  | Nanguoli | Jingbaili | Ruanerli | Korla pear | Chili | Dangshan Suli | Yali | Shuihongxiao |
| --- | --- | --- | --- | --- | --- | --- | --- | --- |
| Fracture | | | | | | | | |
| Cold storage 2 M-0 d | 42.9 | 28.7 | 39.7 | 17.3 | 21.4 | 18.6 | 21.5 | 22.8 |
| Cold storage 2 M-7 d | 6.9 | 5.8 | 5.3 | 17.8 | 22.1 | 16.4 | 19.4 | 21.2 |
| Cold storage 2 M-14 d | 3.1 | 3.8 | - | 20.2 | 23.9 | 16.6 | 20.1 | 25.3 |
| Cold storage 4 M-0 d | 39.8 | 22.5 | 20.9 | 15.2 | 20.1 | 13.2 | 13.7 | 17.3 |
| Cold storage 4 M-7 d | 4.2 | 8.2 | 13.3 | 18.3 | 22 | 17 | 18.5 | 20.3 |
| Cold storage 4 M-14 d | 2.9 | 8 | - | 17.3 | 19 | 15.9 | 19.1 | 18.5 |
| Cold storage 6 M-0 d | 37.7 | 17.1 | 27.4 | 16.6 | 17.5 | 11.9 | 15.6 | 16.6 |
| Cold storage 6 M-7 d | 4.1 | 9.6 | 9.7 | 17.4 | 19.1 | 14.6 | 19.6 | 19.3 |
| Cold storage 6 M-14 d | 2.6 | 10.4 | - | 17.5 | 23.2 | 11.9 | 19.4 | 19.1 |
| Flesh firmness | | | | | | | | |
| Cold storage 2 M-0 d | 51 | 32.4 | 38 | 18.7 | 24.4 | 20.3 | 23.2 | 25.7 |
| Cold storage 2 M-7 d | 7.2 | 6.8 | 5.4 | 19.8 | 24.6 | 17.2 | 21.7 | 23.3 |
| Cold storage 2 M-14 d | 3.2 | 3.8 | - | 22.3 | 29.6 | 17.8 | 22.1 | 26.6 |
| Cold storage 4 M-0 d | 44.8 | 18.4 | 21.4 | 16.7 | 22.4 | 14.3 | 15 | 18.5 |
| Cold storage 4 M-7 d | 4.2 | 8.8 | 13.9 | 19.5 | 27.8 | 16.6 | 21 | 21 |
| Cold storage 4 M-14 d | 2.9 | 9.1 | - | 18.7 | 24.5 | 16.3 | 20.7 | 19.8 |
| Cold storage 6 M-0 d | 43.4 | 14.5 | 23.7 | 17.5 | 19.7 | 13.3 | 16.8 | 18.1 |
| Cold storage 6 M-7 d | 4.3 | 11.2 | 11.6 | 18 | 21.1 | 15.3 | 20.7 | 21.3 |
| Cold storage 6 M-14 d | 2.7 | 10.9 | - | 18.1 | 26.5 | 14.5 | 21.5 | 20.9 |
| F1/F2 | | | | | | | | |
| Cold storage 2 M-0 d | 1.49 | 1.57 | 1.17 | 1.63 | 1.48 | 1.34 | 1.22 | 1.49 |
| Table S3 (continue) | | | | | | | | |
| Cold storage 2 M-7 d | 1.41 | 2 | 1.93 | 1.51 | 1.38 | 1.32 | 1.4 | 1.44 |
| Cold storage 2 M-14 d | 1.68 | 1.73 | - | 1.53 | 1.64 | 1.45 | 1.32 | 1.56 |
| Cold storage 4 M-0 d | 1.39 | 1.24 | 1.28 | 1.39 | 1.38 | 1.21 | 1.26 | 1.25 |
| Cold storage 4 M-7 d | 1.62 | 0.71 | 1.45 | 1.4 | 1.59 | 1.15 | 1.29 | 1.27 |
| Cold storage 4 M-14 d | 1.81 | 1.68 | - | 1.56 | 1.61 | 1.28 | 1.25 | 1.33 |
| Cold storage 6 M-0 d | 1.5 | 1.34 | 1.19 | 1.33 | 1.4 | 1.17 | 1.16 | 1.19 |
| Cold storage 6 M-7 d | 1.54 | 1.58 | 1.45 | 1.36 | 1.52 | 1.15 | 1.26 | 1.3 |
| Cold storage 6 M-14 d | 1.69 | 1.58 | - | 1.52 | 1.42 | 1.21 | 1.27 | 1.26 |
| Adhesiveness | | | | | | | | |
| Cold storage 2 M-0 d | 0.205 | 0.183 | 0.242 | 0.267 | 0.212 | 0.227 | 1.107 | 0.368 |
| Cold storage 2 M-7 d | 0.711 | 0.591 | 0.438 | 0.621 | 0.618 | 0.495 | 0.478 | 0.402 |
| Cold storage 2 M-14 d | 0.552 | 0.732 | - | 0.243 | 0.381 | 0.589 | 0.604 | 0.244 |
| Cold storage 4 M-0 d | 0.135 | 0.158 | 0.205 | 0.17 | 0.166 | 0.282 | 0.214 | 0.267 |
| Cold storage 4 M-7 d | 0.48 | 0.598 | 0.642 | 0.201 | 0.165 | 0.754 | 0.756 | 0.566 |
| Cold storage 4 M-14 d | 0.363 | 0.409 | - | 0.184 | 0.201 | 0.529 | 0.316 | 0.658 |
| Cold storage 6 M-0 d | 0.139 | 0.143 | 0.15 | 0.192 | 0.188 | 0.236 | 0.262 | 0.204 |
| Cold storage 6 M-7 d | 0.145 | 0.144 | 0.157 | 0.18 | 0.157 | 0.186 | 0.205 | 0.183 |
| Cold storage 6 M-14 d | 0.173 | 0.156 | - | 0.333 | 0.187 | 0.196 | 0.153 | 0.178 |
| Cohesiveness | | | | | | | | |
| Cold storage 2 M-0 d | 0.124 | 0.109 | 0.134 | 0.095 | 0.101 | 0.115 | 0.13 | 0.115 |
| Cold storage 2 M-7 d | 0.1 | 0.108 | 0.088 | 0.112 | 0.117 | 0.12 | 0.114 | 0.113 |
| Cold storage 2 M-14 d | 0.088 | 0.084 | - | 0.097 | 0.099 | 0.101 | 0.11 | 0.1 |
| Cold storage 4 M-0 d | 0.118 | 0.136 | 0.124 | 0.105 | 0.104 | 0.127 | 0.111 | 0.121 |
| Cold storage 4 M-7 d | 0.113 | 0.097 | 0.129 | 0.104 | 0.098 | 0.154 | 0.123 | 0.13 |
| Cold storage 4 M-14 d | 0.077 | 0.071 | - | 0.095 | 0.094 | 0.135 | 0.117 | 0.125 |
| Table S3 (continue) | | | | | | | | |
| Cold storage 6 M-0 d | 0.107 | 0.116 | 0.154 | 0.108 | 0.102 | 0.137 | 0.122 | 0.128 |
| Cold storage 6 M-7 d | 0.065 | 0.082 | 0.101 | 0.104 | 0.093 | 0.157 | 0.107 | 0.123 |
| Cold storage 6 M-14 d | 0.096 | 0.083 | - | 0.105 | 0.103 | 0.14 | 0.112 | 0.115 |
| Springiness | | | | | | | | |
| Cold storage 2 M-0 d | 1.557 | 1.432 | 1.607 | 1.061 | 1.157 | 1.241 | 1.192 | 1.327 |
| Cold storage 2 M-7 d | 0.562 | 0.744 | 0.412 | 1.238 | 1.273 | 1.229 | 1.201 | 1.264 |
| Cold storage 2 M-14 d | 0.103 | 0.178 | - | 1.113 | 1.088 | 1.026 | 1.119 | 1.208 |
| Cold storage 4 M-0 d | 1.532 | 1.327 | 1.338 | 1.076 | 1.112 | 1.167 | 1.042 | 1.186 |
| Cold storage 4 M-7 d | 0.397 | 0.741 | 1.095 | 1.138 | 1.121 | 1.458 | 1.267 | 1.406 |
| Cold storage 4 M-14 d | 1.476 | 1.195 | 1.584 | 1.099 | 1.114 | 1.202 | 1.145 | 1.266 |
| Cold storage 6 M-0 d | 0.271 | 0.773 | 0.959 | 1.106 | 1.081 | 1.372 | 1.121 | 1.292 |
| Cold storage 6 M-7 d | 0.101 | 0.824 | - | 1.13 | 1.202 | 1.199 | 1.194 | 1.22 |
| Gumminess | | | | | | | | |
| Cold storage 2 M-0 d | 6.31 | 3.53 | 5.09 | 1.8 | 2.48 | 2.33 | 3.03 | 2.94 |
| Cold storage 2 M-7 d | 0.72 | 0.74 | 0.47 | 2.22 | 2.86 | 2.07 | 2.47 | 2.62 |
| Cold storage 2 M-14 d | 0.28 | 0.32 | - | 2.17 | 2.94 | 1.78 | 2.41 | 2.68 |
| Cold storage 4 M-0 d | 5.28 | 2.52 | 2.68 | 1.77 | 2.32 | 1.83 | 1.66 | 2.25 |
| Cold storage 4 M-7 d | 0.48 | 0.85 | 1.79 | 2.02 | 2.73 | 2.55 | 2.58 | 2.75 |
| Cold storage 4 M-14 d | 0.22 | 0.64 | - | 1.78 | 2.29 | 2.19 | 2.41 | 2.47 |
| Cold storage 6 M-0 d | 4.65 | 1.69 | 3.68 | 1.9 | 1.98 | 1.83 | 2.04 | 2.32 |
| Cold storage 6 M-7 d | 0.27 | 0.93 | 1.18 | 1.85 | 1.96 | 2.42 | 2.21 | 2.61 |
| Cold storage 6 M-14 d | 0.23 | 0.93 | - | 1.89 | 2.74 | 2.03 | 2.41 | 2.41 |
| Chewiness | | | | | | | | |
| Cold storage 2 M-0 d | 9.9 | 5.09 | 8.258 | 1.962 | 2.913 | 2.913 | 3.633 | 3.943 |
| Cold storage 2 M-7 d | 0.445 | 0.663 | 0.197 | 2.774 | 3.651 | 2.553 | 2.983 | 3.356 |
| Table S3 (continue) | | | | | | | | |
| Cold storage 2 M-14 d | 0.029 | 0.057 | - | 2.449 | 3.215 | 1.871 | 2.7 | 3.259 |
| Cold storage 4 M-0 d | 8.158 | 3.526 | 3.726 | 1.919 | 2.581 | 2.146 | 1.743 | 2.677 |
| Cold storage 4 M-7 d | 0.284 | 0.654 | 2.029 | 2.307 | 3.063 | 3.742 | 3.288 | 2.898 |
| Cold storage 4 M-14 d | 0.019 | 0.375 | - | 1.905 | 2.351 | 2.715 | 2.828 | 3.192 |
| Cold storage 6 M-0 d | 6.883 | 2.142 | 5.912 | 2.134 | 2.253 | 2.222 | 2.353 | 2.972 |
| Cold storage 6 M-7 d | 0.084 | 0.796 | 1.198 | 2.058 | 2.128 | 3.416 | 2.514 | 3.429 |
| Cold storage 6 M-14 d | 0.027 | 0.895 | - | 2.14 | 3.352 | 2.445 | 2.887 | 2.979 |

Note: - represents the data was empty.

Table S4

OPLS-DA VIP score of soft-flesh pears after 2 M and 6 M cold-storage, 4 M and 6 M cold-storage.

| Traits | 2 M and 6 M cold-storage | 4 M and 6 M cold-storage |
| --- | --- | --- |
| SSC | 0.59 | 0.06 |
| sucrose | 2.03 | 1.36 |
| sorbitol | 1.59 | 0.23 |
| fructose | 0.06 | 1.33 |
| glucose | 1.24 | 1.16 |
| F/G | 1.10 | 0.45 |
| TSI | 1.08 | 1.83 |
| Quinic acid | 0.72 | 0.81 |
| Shikimic acid | 0.24 | 0.50 |
| Malic acid | 0.02 | 0.42 |
| Citric acid | 1.20 | 1.05 |
| TSS/TA | 1.39 | 1.81 |
| Fracture | 0.37 | 0.01 |
| Table S4 (continue) | | |
| Flesh firmness | 0.45 | 0.03 |
| F1/F2 | 0.27 | 0.23 |
| Adhesiveness | 2.01 | 2.39 |
| Cohesiveness | 0.28 | 0.44 |
| Springiness | 0.06 | 0.06 |
| Gumminess | 0.54 | 0.09 |
| Chewiness | 0.57 | 0.02 |


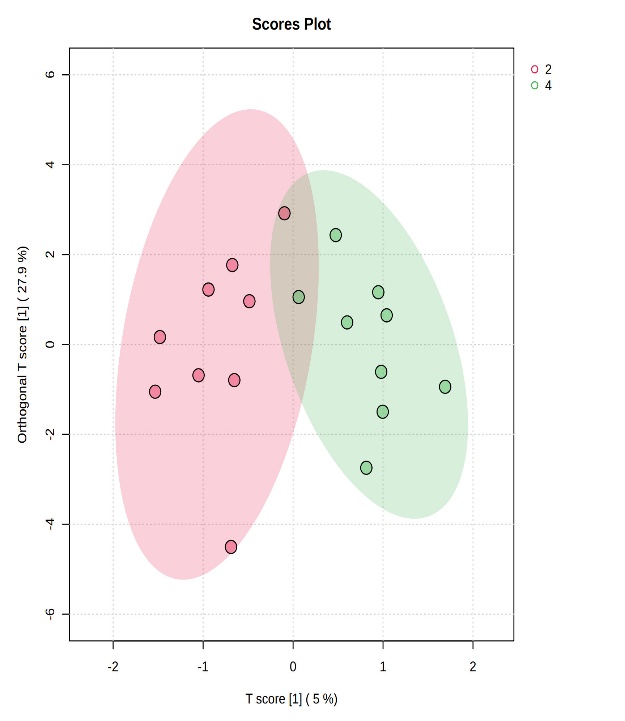


(A)


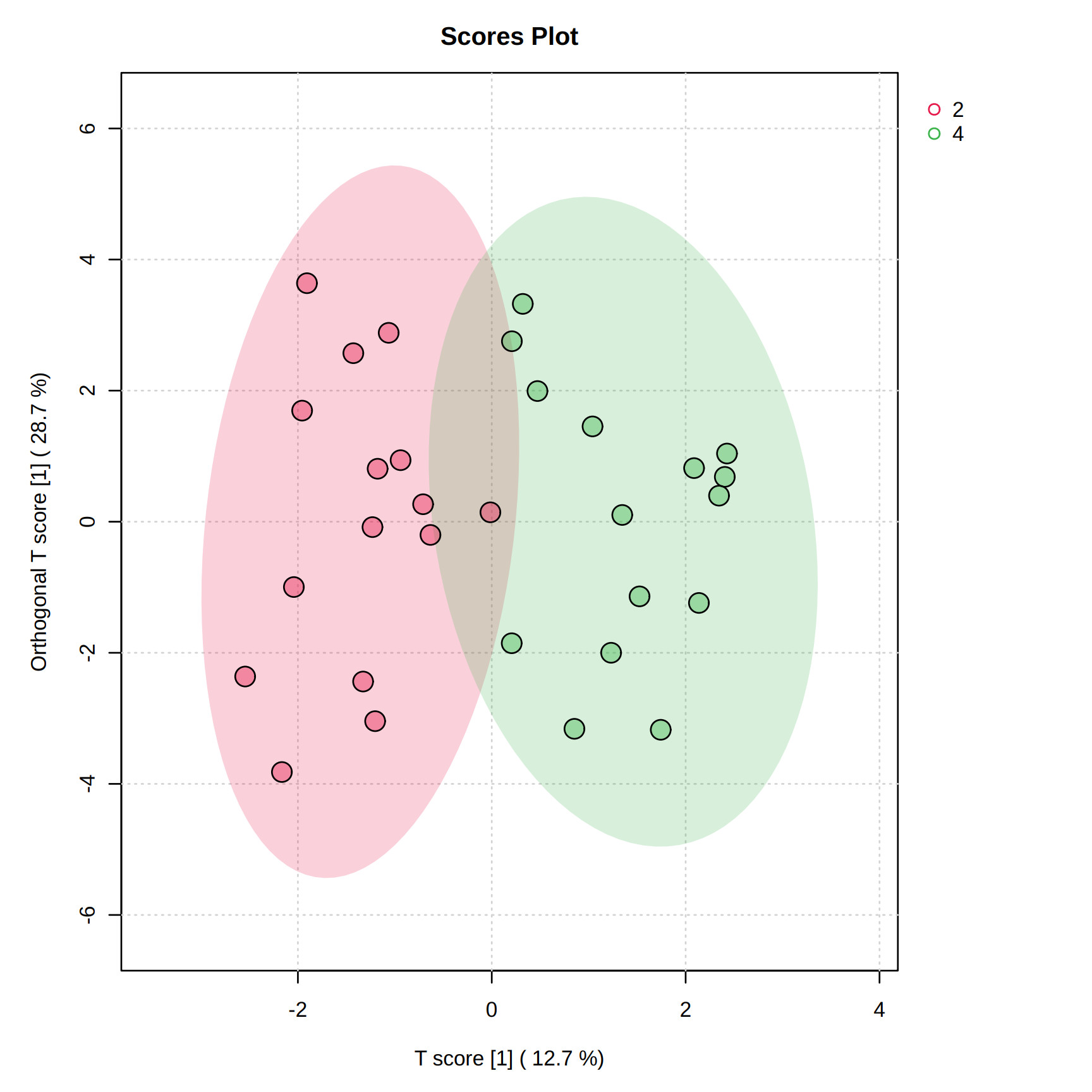


(B)


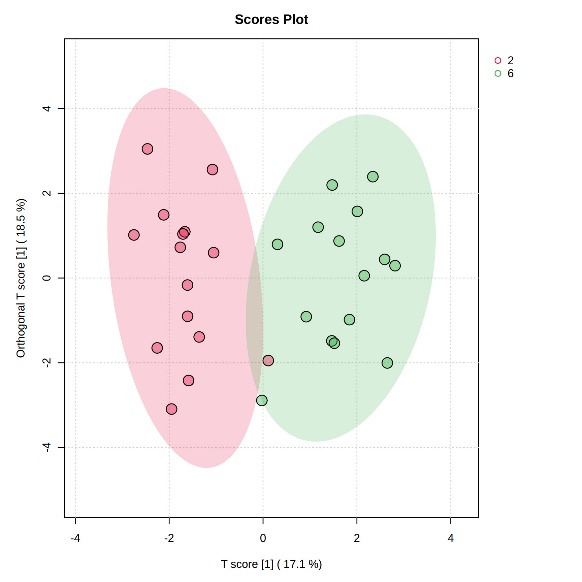


(C)


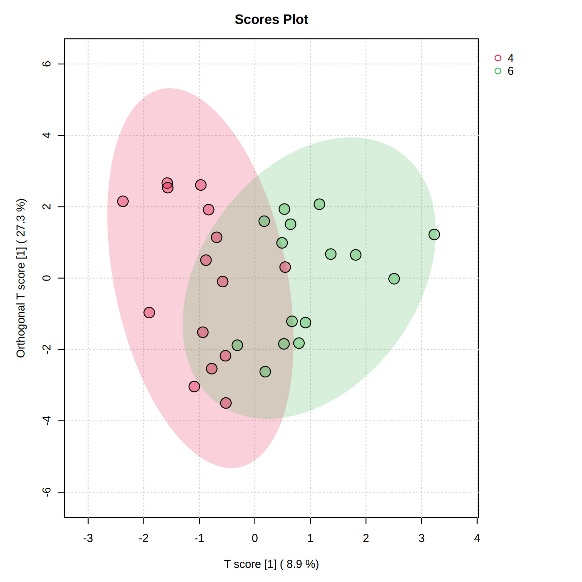


(D)

Fig. S1. OPLS-DA heatmap of pears after different cold-storage times. OPLS-DA of soft-flesh pears after 2M and 4M cold-storage (A). OPLS-DA of crispy-flesh pears after 2M and 4M cold-storage (B). OPLS-DA of crispy-flesh pears after 2M and 6M cold-storage (C). OPLS-DA of crispy-flesh pears after 4M and 6M cold-storage (D).
